# Antitussive, anti-pyretic and toxicological evaluation of Ma-Xing-Gan-Shi-Tang in rodents

**DOI:** 10.1186/s12906-016-1440-2

**Published:** 2016-11-10

**Authors:** Yu-Chin Lin, Ching-Wen Chang, Chi-Rei Wu

**Affiliations:** 1 Department of Medicinal Botanicals and Health Applications, DaYeh University, No.168, University Road, Dacun, Changhua 51591 Taiwan, ROC; 2 Department of Cosmetic Applications and Management, Mackay Junior College of Medicine, Nursing and Management, No. 92, Shengjing Road, Beitou District, Taipei, 11260 Taiwan, ROC; 3 Department of Chinese Pharmaceutical Sciences and Chinese Medicine Resources, China Medical University, No. 91, Hsieh Shih Road, Taichung, 40402 Taiwan, ROC

**Keywords:** Ma-Xing-Gan-Shi-Tang, Antitussive, Anti-pyretic effects, Subacute toxicology test

## Abstract

**Background:**

Ma-Xing-Gan-Shi-Tang (abbreviated as MXGST), an important Chinese herbal prescribed for cough, bronchial inflammation and fever from pneumonia, consists of four medicinal herbs, including Ephedrae herb, Semen Pruni Armeniacae, licorice and Gypsum. These components, especially Ephedrae and Semen Pruni Armeniacae, possess antitussive activities, but they have severe adverse effects.

**Methods:**

The pharmacological activities of MXGST extract in clinical use were investigated with citric acid-induced cough, acetylcholine/histamine-induced bronchial contraction and lipopolysaccharide (LPS)-induced fever in rodents. The subacute toxicology of MXGST extract was evaluated after a 28-day repeated oral administration in rats.

**Results:**

Each gram of MXGST extract contained 60 ± 8 μg of ephedrine, 480 ± 40 μg of glycyrrhizic acid and 440 ± 8 μg of amygdalin according to high performance liquid chromatography and a photodiode array detector. MXGST extract produced pronounced, dose-dependent antitussive effects in guinea pigs and reduced hyperthermic syndrome induced by LPS in rats. MXGST extract blocked the bronchial contraction induced by acetylcholine/histamine. Oral administration of MXGST extract for 28 days did not cause any hematological, biochemical or histological changes in rats.

**Conclusions:**

MXGST extract is a safer, more effective Chinese prescription with antitussive and anti-pyretic effects. The antitussive mechanism of MXGST is related to partially relaxing the bronchial smooth muscle by blocking acetylcholinergic and histaminergic receptors.

## Background

Cough is a beneficial, important protective reflex against irritants or foreign materials, and coughing helps keep the respiratory tract clear. However, cough is a primary symptom of respiratory illness with a high worldwide incidence. It is not only a common symptom of lung diseases, such as upper respiratory tract infection, it is one of the most common complaints that diminishes the patient’s comfort and causes sleep disturbances [1, 2]. Based on the physiological mechanism of the medulla oblongata controlling the coughing reflex, centrally acting antitussive drugs, such as codeine and dextromethorphan, were developed, but their clinic utility has been limited by their undesirable and intolerable side effects such as sedation, nausea, addictive potential and constipation [3, 4]. Therefore, the discovery of novel, safe and effective antitussive agents for treating cough could be facilitated by investigating medicinal plants or herb prescriptions. Ma-Xing-Gan-Shi-Tang (abbreviated as MXGST), an important Chinese herbal medicine for treating cough and fever from pneumonia, consists of Ephedrae (the stem of *Ephedra intermedia*), Semen Pruni Armeniacae (the semen of *Prunus armeniaca*), licorice (the roots of *Glycyrrhiza uralensis*), and Gypsum [5]. Pharmacological reports have indicated that MXGST possesses anti-asthmatic and anti-pyretic activities in allergy-induced or ovalbumin-induced rodent bronchial inflammation models and a rabbit fever model [6–10]. Its major component, Ephedrae, a plant containing multiple active alkaloids, possesses antitussive and anti-asthmatic activities and has been used to treat upper respiratory tract infection or asthma for decades. However, Ephedrae prescriptions are restricted by the US Food and Drug Administration due to their cardiovascular and CNS stimulatory adverse effects, which result from its principal alkaloid ephedrine, a sympathomimetic agonist at α- and β-adrenergic receptors [11, 12]. The second important component Semen Pruni Armeniacae also possesses antitussive activities via its major ingredient amygdalin which biotransformed into cyanide. However, the therapeutic index of cyanide is very narrow and cyanide intoxication can occur [13, 14]. Therefore, the present study attempted to investigate the antitussive and anti-pyretic effects of MXGST extract in rodents with citric acid-induced cough [15] and lipopolysaccharide (LPS)-induced hyperthermia [16]. Then, we further investigated the antitussive mechanism of MXGST extract using acetylcholine/histamine-induced bronchial contraction in guinea pigs. Furthermore, we performed a 28-day repeated oral subacute toxicology test of MXGST extract because the major components of MXGST extract, including Ephedrae, Semen Pruni Armeniacae and licorice have toxicological reports in animal and clinical application [11–14, 17–20].

## Methods

### Preparation of plant extract

Ephedrae (TWU-Plantec-MXGST-0001), Semen Pruni Armeniacae (TWU-Plantec-MXGST-0002), licorice (TWU-Plantec-MXGST-0003) and Gypsum (TWU-Plantec-MXGST-0004) were purchased from the Taiwan market and identified by Professor Lin Y. C. using macroscopic and microscopic methods. These medicinal materials were deposited in the Department of Medicinal Botanicals and Health Applications, DaYeh University. MXGST (8.5 kg) consisted of the aforementioned medicinal materials at a ratio of 4:3:2:8. They were cut into thin slices and place into a container (100 × 100 × 100 cm^3^). Then, distilled water was added and heated at 100 °C for 60 min. The solution was concentrated and dried with a rotary evaporator (Laborota 20 compact, Heidolph Instruments GmbH & Co. (Schwabach, Germany)) at 50 °C and 120–180 mbar. MXGST extract (459 g, 5.4 % yield) was ground into powder, and sealed in a Pyrex glass bottle. The ephedrine, glycyrrhizic acid and amygdalin levels in MXGST extract were approximately 60 ± 8, 480 ± 40 and 440 ± 8 μg/g dry weight, which is in accordance with the calibration curve of ephedrine, glycyrrhizic acid and amygdalin determined by high performance liquid chromatography and a photodiode array detector at 254 and 214 nm. The validation parameters of ephedrine, glycyrrhizic acid and amygdalin are shown in Table 1.

**Table 1:** Validation parameters for ephedrine, glycyrrhizic acid and amygdalin by high performance liquid chromatography

Compound	Concentration range (μg/mL)	R^2^	Linear regression	Intra-D (%)	Inter-D (%)
Ephedrine	6.25–100	0.997	Y = 0.454x-0.0356	1.00–2.74	0.98–3.02
Glycyrrhizic acid	6.25–100	0.999	Y = 0.052x + 0.0046	0.34–3.76	0.62–2.84
Amygdalin	6.25–100	0.990	Y = 0.0645x + 0.0788	1.61–4.33	1.48–3.67

MXGST extract (0.2, 0.4, 1.0 and 2.0 g/kg) was dissolved in sterile distilled water and administered orally 120 min prior to the injection of an inducer. The control was treated with sterile distilled water in the same experiments. Aminophylline (125 mg/kg) was prepared as a suspension with 0.5 % carboxymethylcellulose and administered orally 30 min prior to the injection of an inducer.

### Animals

Male Sprague-Dawley rats (200–250 g) were used in an LPS-induced hyperthermic test and 28-day repeated oral subacute toxicity test. Male guinea pigs (150–200 g) were used in citric acid-induced cough test. The experimental protocol (Protocol No. 102-103-NH) was approved by the Institutional Animal Care and Use Committee (IACUC) of China Medical University and animal care was performed according to the Guiding Principles for the Care and Use of Laboratory Animals. The animals were housed for at least 1 week before starting the experiment in a temperature-(23 ± 1 °C) and humidity-(60 %) regulated environment; they had with free access to standard food in pellets and tap water on a 12–12 h light/dark cycle (light phase: 08:00–20:00 h). After 1 week of acclimatization, eight rodents in each group of the below experiments were used. Then, the drugs were administered and antitussive and anti-pyretic assays were performed using a double-blind method. After behavioral measurement, all animals were killed with carbon dioxide.

### Citric acid-induced cough in guinea pigs

Cough was induced by inhalation of 17.5 % citric acid aerosol, which has been shown to induce the cough reflex in guinea pigs [15]. The aerosol was administered to animals via a small-volume ultrasonic nebulizer that was connected to the bias flow port immediately before the exposure chamber inlet. A volume of 5 mL of citric acid solution was placed into the ultrasonic nebulizer. During the 5-min aerosolization period, approximately 0.4 mL of the solution was nebulized. The onset and frequency of cough during 15 min (aerosolization time + observation time) were recorded.

### Acetylcholine/histamine-induced bronchial contraction in guinea pigs

To explore the antitussive mechanism of MXGST extract, guinea pigs were placed in a glass chamber (3 L volume) and sprayed with a mixture of 0.1 % histamine and 2 % acetylcholine chloride (1:1, v/v) for 15 s. The onset of respiratory distress and tumble (pre-convulsive time) were recorded. The guinea pigs with a pre-convulsive time of more than 120 s were considered insensitive and were discarded. The eligible guinea pigs were randomly divided into five groups, normal, three doses of MXGST extract (0.2, 0.4 and 1.0 g/kg) and aminophylline (125 mg/kg). The pre-convulsive times for each guinea pig within 6 min were observed.

### LPS-induced hyperthermia in rats

The anti-pyretic activity of MXGST extract was evaluated against LPS-induced hyperthermia in rats [16]. Fever was induced by intraperitoneal injection of 10 mL/kg of 100 μg/kg LPS. The rectal temperature was recorded using clinical thermometer immediately before MXGST treatment and immediately before and 1–6 h after LPS injection. Prior to the experiment, the rats were maintained in separate cages for 7 days and the animals with an approximately constant rectal temperature were selected for the study.

### Subacute toxicity study in the rats

The 28-day repeated oral toxicity studies were performed in rats according to the OECD test guideline 407 [21]. Rats were randomly divided into 4 groups of 8 animals each. After an overnight fast, group 1 received sterile distilled water, while groups 2–4 included rats that received MXGST extract at doses of 0.4, 1.0 and 2.0 g/kg body weight, respectively. MXGST extract were administered daily by oral gavage in the volume of 10 mL/kg body weight, once daily for 28 days. The rats were observed daily for any abnormal clinical signs and death during the study period. Body weight and food intake were measured and recorded daily during the study period. At the end of the study, all animals were fasted overnight. The animals were weighed. Blood was collected using the retro-orbital technique with or without ethylenediaminetetraacetic acid (EDTA) for hematological and biochemical analyses, respectively. The animals were sacrificed and body organs, including the lung, liver and kidney, were removed for detailed weight and histopathological changes.

### Hematological parameters and biochemical estimations

Red blood cells (RBCs), white blood cells (WBCs), hematocrit (HCT), hemoglobin (HGB), mean corpuscular hemoglobin (MCH), mean corpuscular hemoglobin concentration (MCHC), mean corpuscular volume (MCV) and platelet counts [22] were determined in control and MXGST extract-treated groups. The serum was carefully aspirated into sample bottles for various biochemical assays. Aspartate transaminase (AST), alanine transaminase (ALT), creatinine, blood glucose, blood urea nitrogen (BUN), total protein and albumin analyses were determined using Radox diagnostic assay kits.

### Organs weight and histology

The rats were quickly dissected and major organs, including the brain, heart, lung, liver, spleen, kidney, adrenal and testis were excised and weighed. The specimens for histopathology were fixed in 10 % neutral, buffered formalin for 18 h at 4 °C. The thickness (4 μm) of each lung, liver and kidney specimen was cut and stained with hematoxylin and eosin stain according to standard laboratory procedures. The stained sections were examined under a microscope for any cellular damage or change in tissue morphology.

### Statistical analysis

All data obtained during antitussive and anti-pyretic activity were expressed in terms of the mean and standard errors, and further analyzed using ANOVA one-way analysis of variance, which was followed by Scheff’s test. When the probability (*p*) was less than 0.05, the difference was considered to be significant.

## Results

### Effects of MXGST extract on citric acid-induced cough in guinea pigs

**Fig. 1:**
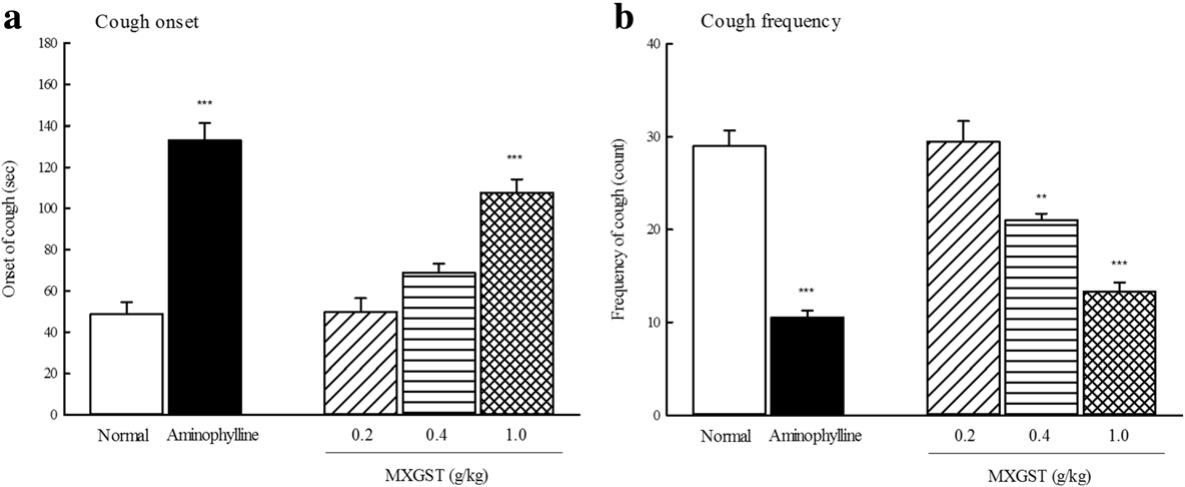
Effect of Ma-Xing-Gan-Shi-Tang (MXGST, 0.2, 0.4, 1.0 g/kg, po) and aminophylline (125 mg/kg, po) on (**a**) the onset time and (**b**) the frequency of citric acid-induced cough response in guinea pig. Each values are represented as mean ± S.E. (*N* = 6). ** *p* < 0.01, *** *p* < 0.001 as compared with the normal group

The onset time for citric acid-induced cough in normal animals ranged from 26.9 to 62.4 s, averaging 48.7 s. The cough incidence in normal animals ranged from 24 to 35 coughs, averaging 29.0. The averaging onset time for citric acid-induced cough in guinea pig treated with MXGST at 0.2, 0.4 and 1.0 g/kg was 49.8, 68.8 and 107.7 s, respectively. The averaging cough frequency in guinea pig treated with MXGST at 0.2, 0.4 and 1.0 g/kg was 29.5, 21.0 and 13.3, respectively. Hence, MXGST extract at 0.4–1.0 g/kg decreased the citric acid-induced cough frequency in a dose-dependent manner (Fig. 1(b); *p* < 0.01, *p* < 0.001), but only at 1.0 g/kg delayed the onset time for citric acid-induced cough (Fig. 1(a); *p* < 0.001). Aminophylline, a positive control, at 125 mg/kg, also decreased the citric acid-induced cough frequency and delayed the onset time for citric acid-induced cough (Fig. 1; *p* < 0.001).

### Effects of MXGST extract on the acetylcholine/histamine-induced bronchial contraction in guinea pigs

**Fig. 2:**
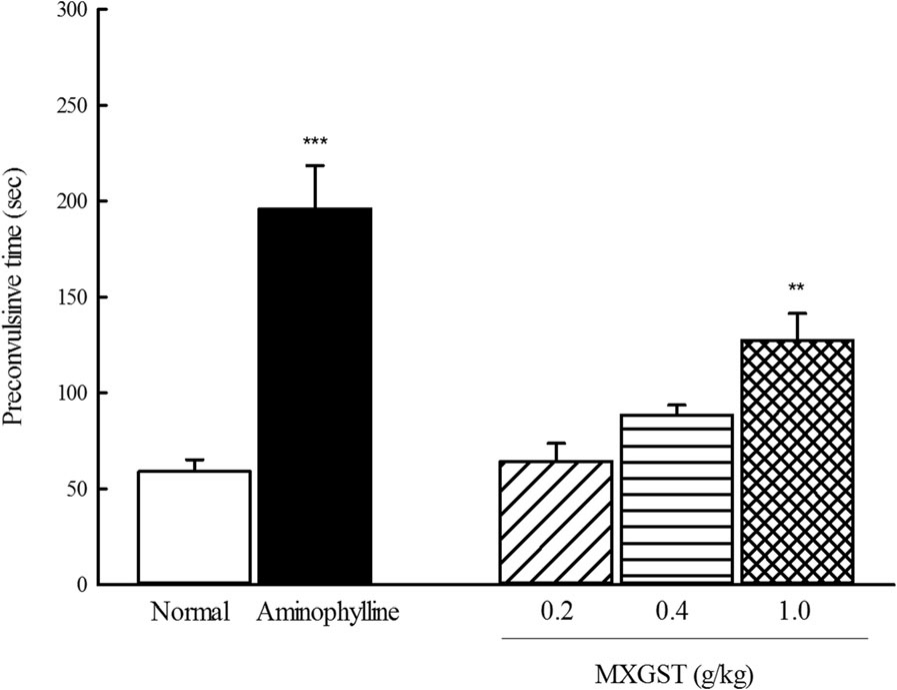
Effect of Ma-Xing-Gan-Shi-Tang (MXGST, 0.2, 0.4, 1.0 g/kg, po) and aminophylline (125 mg/kg, po) on the pre-convulsive time of acetylcholine/serotonin-induced tracheobronchial tonic contraction in guinea pig. Each values are represented as mean ± S.E. (*N* = 6). ** *p* < 0.01, *** *p* < 0.001 as compared with the normal group

The pre-convulsive time of acetylcholine/histamine induced tracheobronchial tonic contraction in guinea pigs ranged from 48.9 to 89.0 s, averaging 59.3 s. The averaging pre-convulsive time of acetylcholine/histamine induced tracheobronchial tonic contraction in guinea pig treated with MXGST at 0.2, 0.4 and 1.0 g/kg was 64.6, 88.5 and 127.6 s, respectively. Hence, MXGST extract (only at 1.0 g/kg) and aminophylline (125 mg/kg) delayed the pre-convulsive time of acetylcholine/histamine induced tracheobronchial tonic contraction (Fig. 2; *p* < 0.01, *p* < 0.001).

### Effects of MXGST extract on LPS-induced hyperthermia in rats

**Fig. 3:**
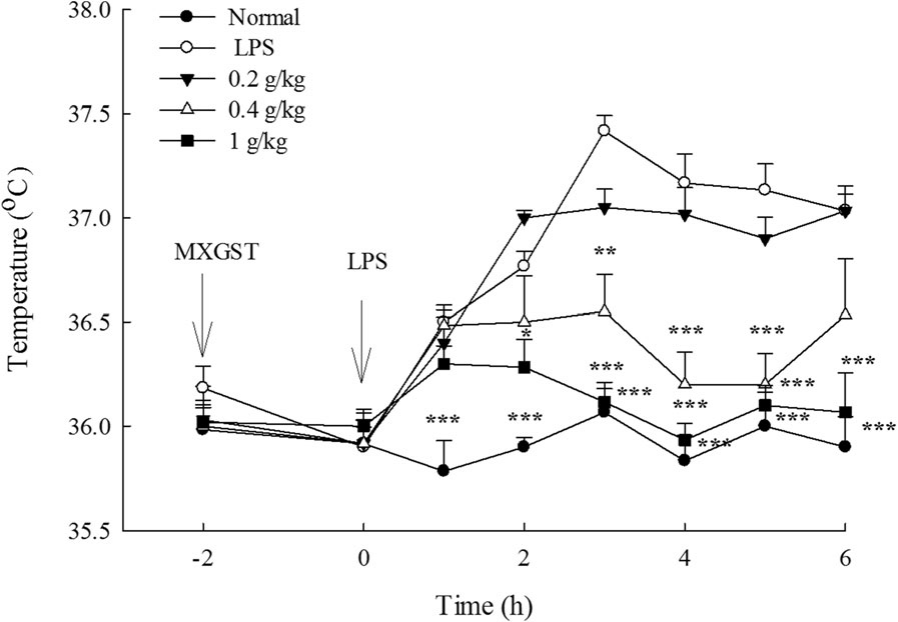
Effect of Ma-Xing-Gan-Shi-Tang (MXGST, 0.2, 0.4, 1.0 g/kg, po) on the lipopolysaccharide (LPS, 100 μg/kg, ip)-induced hyperthermia in rats. Each values are represented as mean ± S.E. (*N* = 6). * *p* < 0.05, ** *p* < 0.01, *** *p* < 0.001, as compared with the LPS group

The rectal temperature of normal rats maintained at 35.8–36.1 °C. The rectal temperature of LPS-induced rats was gradually raised from 35.9 to 37.4 °C, and then maintained at 37.1 °C from 4 to 6 h after LPS injection. So, the rats showed a stable increase in the rectal temperature immediately after LPS injection and reached a plateau period in which their rectal temperature increased approximately 1.5 °C on average, 3 h after LPS injection (Fig. 3; *p* < 0.001). The tendency of rectal temperature raised by LPS in rats treated with MXGST at 0.2 g/kg was similar to that in LPS-induced rats, and finally their rectal temperature maintained at 37.0 °C. Treatment with MXGST at 0.4 g/kg, their rectal temperature was raised to 36.5 °C 1 h after LPS injection, and then maintained at 36.2–36.5 °C. Treatment with MXGST at 1.0 g/kg, their rectal temperature was raised to 36.3 °C 1 h after LPS injection, and then maintained at 35.9–36.1 °C. Hence, MXGST extract, at 0.4 and 1.0 g/kg, produced significant anti-pyretic activity from 2 to 6 h after LPS injection in a dose-dependent manner (Fig. 3; *p* < 0.05 and *p* < 0.01).

### Sub-acute toxicology of MXGST extract

**Fig. 4:**
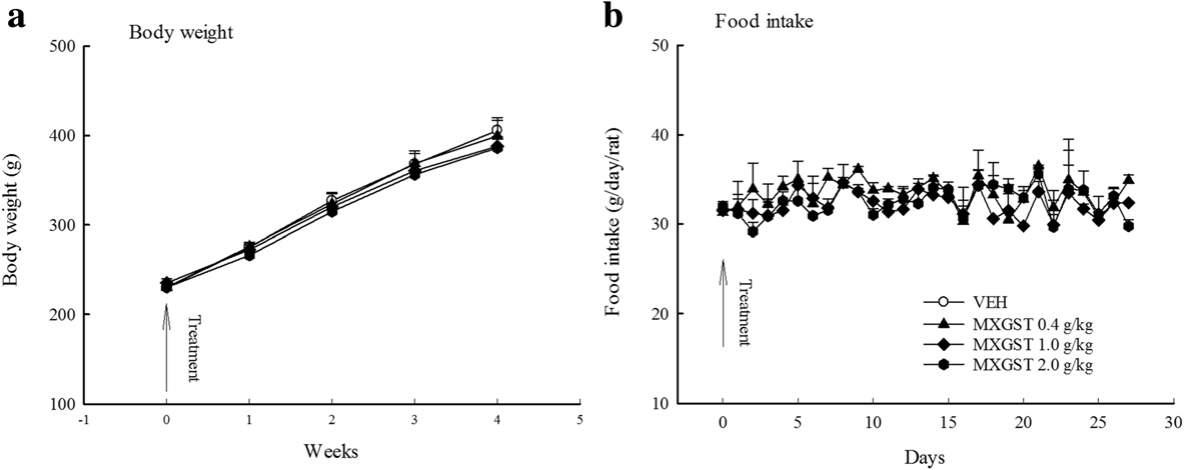
Effect of Ma-Xing-Gan-Shi-Tang (MXGST, 0.4, 1.0, 2.0 g/kg, po) on (**a**) the tendency of body weight and (**b**) daily food intake during 28-day repeated treatment in rats. Each value are represented as mean ± S.E. (*N* = 6)

**Fig. 5:**
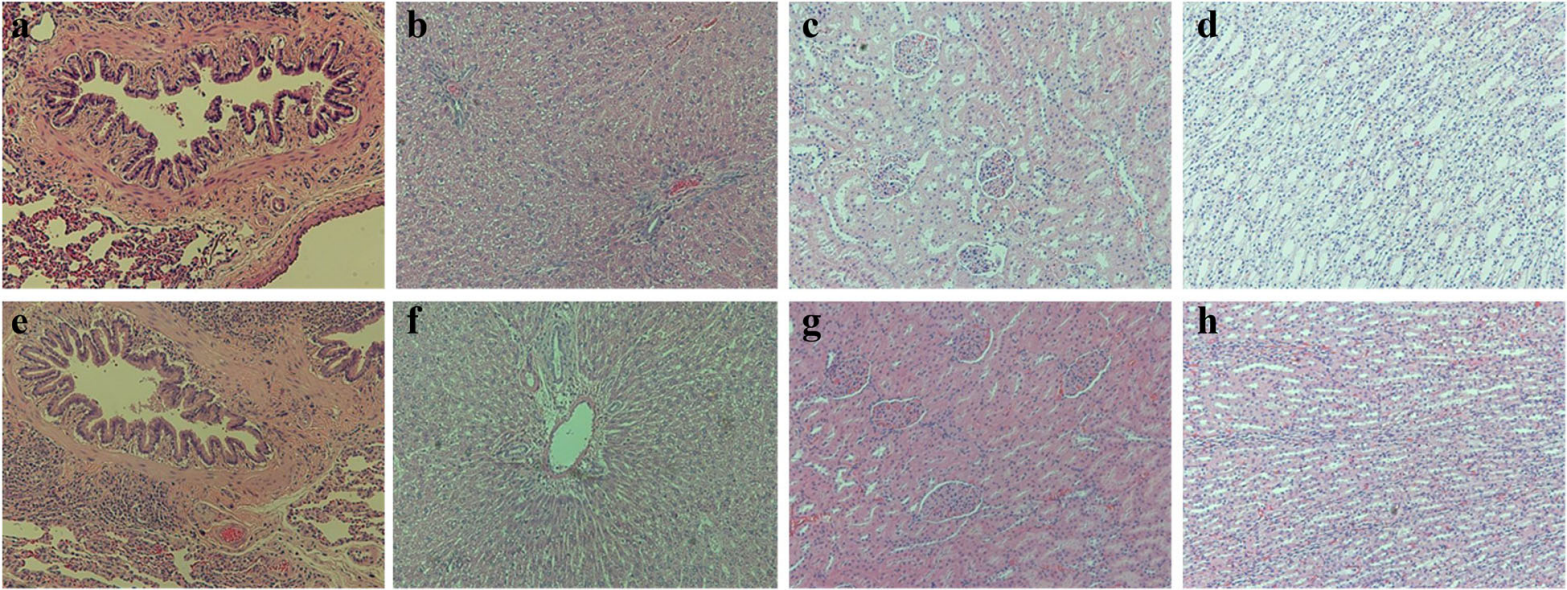
Histology of lung, liver and kidney (H&E, 100x) in rats. (**a**), (**c**), (**e**) and (**i**) Section of lung, liver and kidney from vehicle-treated rats; (**b**), (**d**), (**f**) and (**j**) Section of liver and kidney from Ma-Xing-Gan-Shi-Tang (MXGST, 2.0 g/kg, po)-treated rats

Average body weight of all rats before any treatment was 230 gm. After eight treatment with vehicle, 0.4, 1.0 or 2.0 g/kg of MXGST, their average body weight of each group was 405.8 ± 14.6, 399.8 ± 17.7, 388.0 ± 9.6, and 386.2 ± 12.7 gm, respectively. Oral treatment with MXGST extract at 1.0 or 2.0 g/kg for 28-days caused a slight tendency in body weight loss in a dose-dependent manner, but this was not significant compared with the control group (Fig. 4(a) and (b)). Again, there were no significant difference in the hematological parameters including RBC, HGB, HCT, MCV, MCH, MCHC, WBC and platelet counts between rats treated with MXGST extract (0.4, 1.0 or 2.0 g/kg) or vehicle (Table 2, *p* > 0.05). The biochemical parameters for liver and kidney function tests, such as AST, ALT, creatinine, BUN, blood glucose, total protein and albumin, of rats treated with MXGST extract (0.4, 1.0 or 2.0 g/kg) were not significantly different from those of the control group (Table 3, *p* > 0.05). The urine parameters, including the volume, pH value, protein and glucose, of rats treated with MXGST extract at 0.4, 1.0 or 2.0 g/kg were not significantly different from those of the control group (Table 4, *p* > 0.05). Finally, there were no significant difference between rats treated with MXGST extract (0.4, 1.0 or 2.0 g/kg) or vehicle in their mean weights and gross examinations of major organs, including the brain, heart, lungs, liver, spleen, kidneys, adrenal glands and testis dissected from all rats (Table 5, *p* > 0.05). When MXGST was administered at a higher dose (2.0 g/kg) for 28 days, no histopathological changes in the lungs, liver or kidneys were observed (Fig. 5).

**Table 2:** Effects of Ma-Xing-Gan-Shi-Tang (MXGST, 0.4, 1.0, 2.0 g/kg, po) after 28-day repeated oral administration on hematological parameters in rats

Groups	RBC (10^6^ cells/μL)	HGB (g/dL)	MCV (fj)	MCH (pg)	MCHC (g/dL)	HCT (%)	WBC (10^3^ cells/μL)	Platelet (10^3^ cells/μL)
Normal	7.1 ± 0.3	14.7 ± 0.4	59.0 ± 0.8	20.9 ± 0.4	35.5 ± 0.8	41.5 ± 1.5	8.9 ± 1.2	784.5 ± 68.6
MXGST 0.4 g/kg	7.0 ± 0.1	15.1 ± 0.2	59.3 ± 1.3	21.6 ± 0.3	36.5 ± 0.6	41.6 ± 1.1	9.2 ± 1.5	816.2 ± 42.3
MXGST 1.0 g/kg	6.8 ± 0.1	14.7 ± 0.2	58.0 ± 1.1	21.6 ± 0.3	37.3 ± 0.5	39.5 ± 1.0	8.0 ± 1.0	839.7 ± 36.8
MXGST 2.0 g/kg	7.3 ± 0.2	15.6 ± 0.3	58.7 ± 1.2	21.5 ± 0.3	36.7 ± 0.5	42.6 ± 1.2	10.9 ± 1.2	936.2 ± 55.9

Data are represented with mean ± SEM, *N* = 6

**Table 3:** Effects of Ma-Xing-Gan-Shi-Tang (MXGST, 0.4, 1.0, 2.0 g/kg, po) after 28-day repeated oral administration on plasma biochemical parameters in rats

Groups	Glu (mg/dL)	TP (mg/dL)	Albumin (mg/dL)	ALT (U/L)	AST (U/L)	BUN (mg/dL)	Creatinine (mg/dL)
Normal	173.8 ± 12.9	5.8 ± 0.4	3.9 ± 0.1	74.1 ± 3.8	73.7 ± 2.7	25.2 ± 2.4	0.29 ± 0.02
MXGST 0.4 g/kg	188.2 ± 3.0	6.2 ± 0.1	3.9 ± 0.1	70.8 ± 5.2	69.3 ± 1.5	27.0 ± 2.4	0.31 ± 0.02
MXGST 1.0 g/kg	185.2 ± 5.4	5.9 ± 0.1	3.8 ± 0.1	65.3 ± 4.0	78.5 ± 6.5	25.3 ± 2.4	0.28 ± 0.02
MXGST 2.0 g/kg	193.0 ± 7.1	6.1 ± 0.2	4.0 ± 0.1	58.5 ± 2.4	71.1 ± 2.2	21.5 ± 1.1	0.30 ± 0.02

Data are represented with mean ± SEM, *N* = 6

**Table 4:** Effects of Ma-Xing-Gan-Shi-Tang (MXGST, 0.4, 1.0, 2.0 g/kg, po) after 28-day repeated oral administration on urine parameters in rats

Groups	Volumes (mL)	pH value	Protein (mg/L)	Glucose (mmol/L)
Normal	22.0 ± 1.9	7.2 ± 0.1	37.1 ± 2.3	6.9 ± 0.8
MXGST 0.4 g/kg	21.8 ± 2.3	7.1 ± 0.2	38.5 ± 2.9	6.8 ± 0.7
MXGST 1.0 g/kg	21.7 ± 3.3	6.9 ± 0.3	44.0 ± 4.7	6.7 ± 1.5
MXGST 2.0 g/kg	20.2 ± 3.3	6.8 ± 0.1	39.5 ± 2.7	6.8 ± 0.8

Data are represented with mean ± SEM, *N* = 6

**Table 5:** Effects of Ma-Xing-Gan-Shi-Tang (MXGST, 0.4, 1.0, 2.0 g/kg, po) after 28-day repeated oral administration on organ weight in rats

Groups	Brain (g)	Heart (g)	Lung (g)	Liver (g)	Spleen (g)	Kidney (g)	Adrenal (mg)
Normal	1.97 ± 0.05	1.37 ± 0.04	1.38 ± 0.09	15.71 ± 0.56	0.80 ± 0.07	2.71 ± 0.09	61 ± 2
MXGST 0.4 g/kg	1.98 ± 0.03	1.39 ± 0.06	1.45 ± 0.04	15.49 ± 0.71	0.97 ± 0.11	2.73 ± 0.10	59 ± 2
MXGST 1.0 g/kg	1.99 ± 0.04	1.26 ± 0.05	1.34 ± 0.02	14.56 ± 0.43	0.81 ± 0.04	2.57 ± 0.03	57 ± 3
MXGST 2.0 g/kg	2.07 ± 0.04	1.33 ± 0.08	1.44 ± 0.08	15.06 ± 0.69	0.92 ± 0.09	2.57 ± 0.11	60 ± 4

Data are represented with mean ± SEM, *N* = 6

## Discussion

MXGST is mainly used by traditional Chinese physicians to treat cough from pneumonia. A recent report indicated that MXGST prolonged the coughing onset and decreased the coughing frequency of ammonia-induced cough in rats [23]. Citric acid-induced cough is a very useful model for assessing the antitussive effects of medicinal plants and synthetic compounds. Hence, we first performed a citric acid-induced cough to evaluate the antitussive effects of MXGST extract in guinea pigs. The dosage (0.4 g/kg) of MXGST was chosen according to the conversion of the clinical daily therapeutic dosage [5] to animal equivalent dosage by body surface area and other reports [6, 7, 23]. The present data are similar to the effects of MXGST on ammonia-induced cough [23], indicating that MXGST and the aminophylline positive control had antitussive effects on the citric acid-induced cough model. Furthermore, citric acid stimulated rapidly adapting, histaminergic and acetylcholinergic receptors that are located in the larynx and upper airways of the tracheobronchial tree by opening the pH gated ion channels to induce cough and tracheobronchial contraction [2]. The inhalation of citric acid also causes bronchial hyperresponsiveness to acetylcholine and hypersensitivity to histamine-induced microvascular leakage, which can then induce tracheobronchial contraction and cough [2]. As a result, the antitussive mechanism of MXGST extract on the bronchial tract was explored by acetylcholine/histamine-induced tracheobronchial contraction in guinea pigs. We also found that MXGST and positive control aminophylline relaxed the tracheobronchial contraction caused by acetylcholine/histamine. Another researcher indicated that MXGST relieved airway resistance increases by *Dermatophagoides Pteronyssinus* in guinea pigs [7]. Moreover, Ephedrae possessed the antitussive effects of citric acid-induced cough in guinea pigs [24]. Additionally, ephedrine and methylephedrine (both active ingredients of Ephedrae) have antitussive and anti-asthmatic effects in animal models and humans. They can dilate bronchial smooth muscle via activating both sympathomimetic α- and β-adrenergic receptors [25, 26]. Amygdalin, a major constituent of Semen Pruni Armeniacae, also possesses antitussive activities via inhibiting the central cough center when it is biotransformed into cyanide, an active ingredient [25]. Licorice also decreased the cough response induced by ammonia or capsaicin [27, 28]. Moreover, co-treatment with Ephedrae and Semen Pruni Armeniacae has better antitussive effects than Ephedrae or Semen Pruni Armeniacae alone [9]. Thus, we suggested that MXGST extract, at a lower dosage than the reported dosage of each medicinal component, possessed antitussive effects. These effects might be due to the synergic effects of its medicinal components, especially of Ephedrae and Semen Pruni Armeniacae [9]. The antitussive mechanism of MXGST might be related to dilating the bronchial smooth muscle by inhibiting the histaminergic and acetylcholinergic receptors in the bronchial tract, activating both sympathomimetic α- and β-adrenergic receptors from ephedra alkaloids and inhibiting the central cough center from amygdalin [25, 26]. However, citric acid-induced cough and tracheobronchial constriction are partially mediated by the nonadrenergic–noncholinergic-nonhistaminergic nervous systems, which include tachykinins, leukotrienes and prostaglandin E_2_ from mast cells [29–33]. Therefore, whether the antitussive mechanism of MXGST might be related to stabilizing mast cells and inhibiting other inflammatory mediators and nervous systems must be investigated in the future.

Second, the anti-pyretic effects of MXGST extract on LPS-induced hyperthermia in rats were evaluated because traditional Chinese physicians mainly use MXGST to treat fever caused by pneumonia. This result is consistent with other report that MXGST possesses anti-pyretic effects on a typhoid/paratyphoid vaccine-induced fever model in rabbits [6]. Some researchers indicated that Gypsum possesses anti-pyretic effects in Brewer’s yeast-induced hyperthermia via decreasing the hypothalamus prostaglandin E_2_ levels [34]. Glycyrrhetic acid, a major ingredient of licorice, possesses anti-pyretic effects in Brewer’s yeast-induced fever in rats via the pituitary adrenal axis [35]. The herb pairs, Ephedrae and Gypsum, significantly attenuated Brewer’s yeast-induced fever in rats [36]. Therefore, it can be suggested that MXGST possesses anti-pyretic effects via the synergic effects of its medicinal components, especially Gypsum and Ephedrae [8, 36], and this effect might be mediated through the modulation of prostaglandin E_2_ synthesis in the hypothalamus adrenal gland axis from glycyrrhizic acid and Gypsum [34, 35].

Each MXGST component has been reported to have the various adverse effects, such as cardiovascular and CNS toxicology of Ephedra [11, 12, 18, 19], edema and mineralocorticoid excess syndrome of licorice [20], cyanide toxic reaction of Semen Pruni Armeniacae [13, 14, 17], and allergic response of Gypsum [37]. Therefore, we further evaluated the subacute toxicology of MXGST at 0.4, 1.0 or 2.0 g/kg after 28-day repeated oral administration in rats because MXGST possesses antitussive and anti-pyretic effects at doses of 0.4–1.0 g/kg. Rats treated with MXGST extract at 0.4, 1.0 or 2.0 g/kg body weight daily for 28 days survived throughout the period and did not show any changes in their general behavior or other physiological activities. We also found that oral administration of the MXGST extract at 5 times the effective dose for 28 days did not cause any toxicological responses or histopathological changes, and then MXGST which consists of Ephedrae, Semen Pruni Armeniacae, licorice and Gypsum did not show any toxicities such as cardiovascular and CNS toxicology from Ephedra [11, 12, 18, 19], mineralocorticoid excess syndrome from licorice [20], cyanide toxic reaction of Semen Pruni Armeniacae [13, 14, 17], and allergic response of Gypsum [37]. Recent reports indicated that Ephedrae decreased the acute toxicology of Semen Pruni Armeniacae, and licorice decreased acute toxicology of Ephedrae when these herb pairs are co-treated [9, 38]. Therefore, we found that MXGST extract has a higher safe therapeutic index because the ephedrine and amygdalin levels in the MXGST extract for the antitussive and anti-pyretic tests are approximately 24–60 μg ephedrine/g body weight and 176–440 μg amygdalin/g body weight (thousandth of LD_50_ dose of ephedrine and amygdalin).

## Conclusion

Based on our present results, MXGST extract is a very safe Chinese prescription that possesses pronounced antitussive and anti-pyretic activities, confirming its clinical utility for cough, bronchial inflammation and fever caused from pneumonia. The antitussive mechanism of MXGST might be related to dilating the bronchial smooth muscle by inhibiting histaminergic and acetylcholinergic receptors in the bronchial tract, activating both sympathomimetic α- and β-adrenergic receptors from ephedra alkaloids and inhibiting central cough center from amygdalin.

## Abbreviations

ALT: Alanine transaminase
AST: Aspartate transaminase
BUN: Blood urea nitrogen
HCT: Hematocrit
HGB: Hemoglobin
LPS: Lipopolysaccharides
MCH: Mean corpuscular hemoglobin
MCHC: Mean corpuscular hemoglobin concentration
MCV: Mean corpuscular volume
MXGST: Ma-Xing-Gan-Shi-Tang
RBC: Red blood cells
WBC: White blood cells
